# Effect of *Crocus sativus* Extract Supplementation in the Metabolic Control of People with Diabetes Mellitus Type 1: A Double-Blind Randomized Placebo-Controlled Trial

**DOI:** 10.3390/nu16132089

**Published:** 2024-06-29

**Authors:** Parthena Giannoulaki, Evangelia Kotzakioulafi, Alexandros Nakas, Zisis Kontoninas, Eleni Karlafti, Polykarpos Evripidou, Konstantinos Kantartzis, Christos Savopoulos, Michail Chourdakis, Triantafyllos Didangelos

**Affiliations:** 1 Department of Clinical Nutrition, University General Hospital of Thessaloniki AHEPA, 54636 Thessaloniki, Greece; 2 Diabetes Center, 1st Propaedeutic Department of Internal Medicine, School of Medicine, University General Hospital of Thessaloniki AHEPA, Aristotle University of Thessaloniki, 54636 Thessaloniki, Greece; ekotzaki@auth.gr (E.K.); al.nakas@hotmail.com (A.N.); drziko2401@gmail.com (Z.K.); linakarlafti@hotmail.com (E.K.); polysevripidou@gmail.com (P.E.); csavvopo@auth.gr (C.S.); didang@auth.gr (T.D.); 3 Department of Internal Medicine IV, Division of Endocrinology, Diabetology and Nephrology, University of Tübingen, 72076 Tübingen, Germany; konstantinos.kantartzis@med.uni-tuebingen.de; 4 Institute for Diabetes Research and Metabolic Diseases (IDM), Helmholtz Centre Munich, University of Tübingen, 72076 Tübingen, Germany; 5 German Center for Diabetes Research (DZD), 72076 Tübingen, Germany; 6 Laboratory of Hygiene, Social & Preventive Medicine and Medical Statistics, School of Medicine, Faculty of Health Sciences, Aristotle University of Thessaloniki, 54124 Thessaloniki, Greece; mhourd@gapps.auth.gr

**Keywords:** diabetes mellitus type 1, continuous glucose monitoring, probiotics, saffron extract, *Crocus sativus* L.

## Abstract

Introduction–Background: Data from experimental trials show that *Crocus sativus* L. (saffron) is considered to improve glycemia, lipid profile, and blood pressure and reduce oxidative stress. So far, clinical trials have been conducted in individuals with metabolic syndrome and Diabetes Mellitus type 2 (DMT-2). The purpose of this study is to assess the effectiveness of saffron in individuals with Diabetes Mellitus type 1 (DMT-1). Patients–Methods: 61 individuals with DMT-1, mean age 48 years old (48.3 ± 14.6), 26 females (42.6%) were randomized to receive a new oral supplement in sachets containing probiotics, prebiotics, magnesium, and *Crocus sativus* L. extract or placebo containing probiotics, prebiotics and magnesium daily for 6 months. Glycemic control was assessed with a continuous glucose monitoring system and laboratory measurement of HbA1c and lipid profile was also examined. Blood pressure at baseline and end of intervention was also measured. Individuals were either on a continuous subcutaneous insulin infusion with an insulin pump or in multiple daily injection regimens. Diabetes distress and satiety were assessed through a questionnaire and body composition was assessed with bioelectrical impedance. Results: At the end of the intervention, the two groups differed significantly only in serum triglycerides (*p* = 0.049). After 6 months of treatment, a significant reduction in the active group was observed in glycated hemoglobin (*p* = 0.046) and serum triglycerides (*p* = 0.021) compared to baseline. The other primary endpoints (glycemic control, lipid profile, blood pressure) did not differ within the groups from baseline to end of intervention, and there was no significant difference between the two groups. Diabetes distress score improved significantly only in the active group (*p* = 0.044), suggesting an overall improvement in diabetes disease burden in these individuals but that was not significant enough between the two groups. Conclusions: A probiotic supplement with saffron extract improves serum triglycerides in well-controlled people with DMT-1 and may potentially be a valuable adjunct for enhancing glycemic control.

## 1. Introduction

Type 1 diabetes mellitus (DMT-1) is a metabolic disorder, mainly of autoimmune origin, characterized by hyperglycemia caused by a complete lack of insulin secretion. Chronic hyperglycemia in DMT-1 can lead to long-term damage and dysfunction in multiple organs, especially the eyes, kidneys, nerves, heart, and blood vessels [1]. As a result of chronic hyperglycemia, oxidative stress deteriorates further the damage to the aforementioned organs [2]. Although optimal control of plasma glucose and lipid concentrations reduces the incidence of diabetes mellitus (DM) complications [3], optimal glycemic control is quite difficult to achieve and maintain over time, especially in patients with DMT-1 due to hypoglycemia induced by incorrect insulin delivery [4].

Moreover, the increasing cost of treating DM and its complications consists of a financial burden for many economies, whereas the quality of life of people with DM-related complications deteriorates, and life expectancy also decreases [5,6]. The total estimated cost of diagnosed diabetes in the USA in 2022 was $412.9 billion [7]. The high cost of DM therapies and their side effects have led to research into contemporary alternative treatments.

Several studies [8,9,10] demonstrate the protective effect of a variety of antioxidant plant products against cell and tissue damage, such as the extract of saffron or *Crocus sativus* L. *Crocus sativus* L. is a bulbous and perennial plant with red stigmas and attached yellowish style [10]. Its red stigmas, in dried form, is the spice commonly known as saffron or crocus which is produced mainly in Greece, Iran, and India [11]. Saffron is characterized by its peculiar features, including its aromatic smell, bitter taste, and intense red color, and contains potential pharmacological active ingredients. Its bitter taste originates from picrocrocin, a,b-D-glucoside of hydroxyl-safranal. The chief components of stigma are crocetin, its glucosidic derivatives, safranal, picrocrocin, crocins and flavonoids like kaempferol and quercetin [10]. The primary active ingredients are crocins (approximately 10% of the total content). High-quality saffron consists of approximately 30% crocins, 5–15% picrocrocin, and often 2.5% volatile compounds, one of which is safranal. Greek saffron, known as Greek red saffron, has the highest concentration of the above ingredients [11].

Studies have shown that the saffron has antidiabetic and antioxidant effects [12,13,14,15,16,17,18,19]. Also, the possibilities of saffron in the treatment of hyperlipidemia, hypertension, atherosclerosis, and myocardial damage have been highlighted in experimental studies [20,21,22,23] and in systematic reviews [11] and meta-analyses [24,25]. Previous systematic reviews on the impact of *Crocus sativus* L. on metabolic profile in patients with DM or metabolic syndrome (MS) demonstrated implausible findings due to the low-quality clinical trials assessed. The available limited evidence (five studies with saffron 15–100 mg daily for 2–3 months, five studies with crocin 5–100 mg for 2–3 months) showed a potentially favorable effect of saffron in fasting blood glucose levels. Fourteen studies were included in the review, and individuals with DMT-1 were included in only one study [11]. A more recent meta-analysis demonstrated improvements in fasting blood glucose (FBG) in the DMT-2 and prediabetes individuals and HbA1c only in the prediabetes individuals [25]. Another meta-analysis in herbs and spices effectiveness in glycemic control in DMT-2 has shown that *Crocus sativus* L. (five studies with saffron 15–400 mg daily (tablet or powder) for 2–3 months and two studies with crocin with 15 mg for 3 months) has significantly reduced fasting blood glucose in individuals with DMT-2 [24].

Probiotics have shown promising results in improving metabolic health, especially in reducing body weight, fasting blood glucose, HbA1c, and insulin in people with DMT-2 [26]. Although a recent randomized controlled trial (RCT) in children with DMT-1 has shown no effect in glycemic control when administering *L. rhamnosus* and *B. lactis* for 6 months [27], evidence from a meta-analysis of RCTs shows that administration of two species of probiotics and more have a significant effect in reducing body weight and HbA1c in people with DMT-2 [26]. Specifically, *B. lactis*, *B. longum*, and *L. rhamnosus* can favor weight loss in people with DMT-2, whereas *B. breve*, *B. longum*, and *L. rhamnosus* have shown a reduction in FBG and *B. breve*, *B. lactis*, and *B. longum* reduce HbA1c significantly [26].

To our knowledge, there is no study in the current literature to investigate the impact of saffron extract on the metabolic profile of individuals with DMT-1. However, because saffron is not marketed in our country as a stand-alone supplement but only in combination with probiotics and prebiotics, we decided to use this combination in our study. The aim of this randomized, double-blind placebo-controlled study was to evaluate the effectiveness of extract of *Crocus sativus* L. on the metabolic control in people with DMT-1 using glucose monitoring systems.

## 2. Materials and Methods

### 2.1. Protocol Registration

The study was registered in clinicaltrials.gov with registration number NCT05933460. The study protocol was submitted and approved by the Bioethics Committee of the School of Medicine of the Aristotle University of Thessaloniki (protocol number 1.46-21/11/2018). The study follows the CONSORT statement for reporting and presenting the results of clinical trials (Supplementary Materials). The study was conducted according to the principles of the Declaration of Helsinki [28].

### 2.2. Sample Size Calculation

The sample size was calculated based on the assumption that the HbA1c at the end of the study will differ by 0.5% between groups with a statistical power of 80% and a statistical significance level of 0.05, 28 patients are required to be included in the study (14 patients in each group).

So far, clinical studies examining the effect of saffron and its components on patients [29,30,31,32] or healthy volunteers [33,34] had a varying sample size from 10 to 80 subjects.

### 2.3. Patients Recruitment

The study was conducted at the Diabetes outpatient clinic of the 1st Propaedeutic Department of Internal Medicine of the University General Hospital of Thessaloniki AHEPA from April 2022 to March 2023. Sixty-one adults with DMT-1, fulfilling the inclusion criteria, were enrolled according to the following inclusion and exclusion criteria.

Inclusion criteria included: (i) adults with DMT-1, (ii) diabetes duration > 12 months, (iii) their diabetes management treatment included Continuous Glucose Monitoring (CGM) or Intermittent Glucose Monitoring (either on Multiple Daily injections or continuous subcutaneous insulin infusion), and (iv) did not intend to change their diabetes therapy. Exclusion criteria were: (i) adults with DMT-2, (ii) adults with liver disease or impaired liver function, (iii) women with DMT-1 planning for pregnancy, pregnant or breastfeeding, (iv) adults with Chronic Kidney Disease with GFR < 60 mL/min/1.73 m^2^, (v) adults on antiplatelet treatment for non-preventive purposes, (vi) people on advanced hybrid closed loop treatment, (vii) adults with history of allergic reaction to *Crocus sativus*, (viii) adults with daily herbal consumption or taking daily multivitamin or probiotic and prebiotic supplements, (ix) adults that were unable to understand the study framework and consent to the trial.

### 2.4. Randomization and Allocation

The allocation and randomization sequences were performed using a table of random numbers that was produced by a computer-generated sequence in blocks of two. The randomization order and treatment allocation were concealed from the responsible researcher and statistician. All tests and measurements were performed by a physician and a dietitian blinded to the allocation of the participants. Supplement containers were administered by a third independent researcher who was unaware of the randomization sequence. Supplement containers were sequentially numbered, packed identically, and dispensed according to the allocation sequence. There were no dropouts, and all patients completed the study.

### 2.5. Intervention Details

People were informed about the trial procedures and signed an informed consent form. Individuals were randomized to receive a new oral supplement in sachets containing probiotics, prebiotics, Magnesium, and *Crocus sativus* L. extract (LactoLevure ProbioMood; UNI-PHARMA S.A., Kifisia, Greece) or placebo daily for 6 months. The exact formulation of the product is presented in Table 1.

Individuals were advised to take 3 sachets per day diluted in 200 mL water each, consisting of a total daily 84 mg *Crocus sativus* extract (3.5% bioactive ingredients such as crocin and safranal). Placebos were provided by the company in identical containers containing all ingredients without the *Crocus sativus* extract. Participants were obliged to return empty containers back for compliance purposes.

### 2.6. Antidiabetic and Concomitant Medication

All participants were using a continuous glucose monitoring system (Guardian™ Sensor 3, Medtronic, Dublin, Ireland) or intermittent glucose monitoring system (FreeStyle Libre, Abbott, Chicago, IL, USA) either on multiple daily injections (MDI) or continuous subcutaneous insulin infusion (CSII). Other comorbidities, diabetes complications, and concomitant medication were recorded as part of the participant’s medical history. Participants were stable throughout the study period and no change was required to their overall treatment regimen.

### 2.7. Data Collection—Measurements and Tests

All laboratory analyses were performed in the Biochemistry Laboratory of the University General Hospital of AHEPA. Primary outcomes were analyzed with the high-performance liquid chromatography (HPLC) method (for HbA1c) and enzymatic colorimetry (total cholesterol, HDL cholesterol, triglycerides, and fasting blood glucose). LDL cholesterol was calculated with the Friedewald equation [35]. Primary outcomes are given in mg/dL. Blood pressure was measured in two consecutive measurements according to current guidelines [36] and then averaged by an experienced physician.

In terms of secondary outcomes, fourteen days’ reports from continuous and intermittent glucose monitoring systems were downloaded both at baseline and at the end of the intervention. Anthropometric measurements (body weight, height, waist circumference) and body composition analysis with bioelectrical impedance (Bodystat 1500, Bodystat Ltd., Douglas, Isle of Man) were performed by an experienced dietitian at all-time points. Furthermore, a scored questionnaire regarding diabetes distress [37,38] was also employed to assess the feeling of disease distress at baseline and after six months of intervention, and a Subjective Satiety and Hunger Rating questionnaire [39] was also used at the end of the intervention.

To neutralize diet and physical activity as the main confounders of the outcomes and ensure the reliability of our results, a weighted food diary of 2 weeks was documented to assess the nutritional intake, both at baseline and at the end of the intervention and analyzed. The food diary was crosschecked with the participant each time with a one-hour session by an experienced dietitian to validate the accuracy of the reporting. Dietary data analysis was performed with Genesis R&D (Version 9.10-2012, ESHA Research Inc., Salem, OR, USA), adapted for Greek food items. The energy and macronutrient intake were averaged and are presented as daily intake in the results. Along with the dietary data, the level of physical activity was assessed with the International Physical Activity Questionnaire (IPAQ) [40] at baseline and after six months of intervention.

### 2.8. Statistical Analysis

Statistical analysis was performed using SPSS v.23 software (IBM Corp., Armonk, NY, USA, Released 2015. IBM SPSS Statistics for Windows, Version 23.0. Armonk, NY, USA: IBM Corp.). Data were collected via a standardized form in Microsoft Excel (Microsoft Corporation, Redmond, WA, USA, 2018. Microsoft Excel, Available at: https://office.microsoft.com/excel (accessed on 25 April 2024)). Data were tested for normal distribution using Shapiro Wilk’s Shapiro test with a significance level of *p* < 0.05. Data are presented with mean and standard deviation if normally distributed and with median and interquartile range if not normally distributed. Categorical data are presented as % frequencies. To test the difference between the 2 groups at baseline, the independent samples *t*-test was used, while the paired samples *t*-test was used to test the difference from baseline to the end of the intervention in each group. The independent samples *t*-test was used to test the difference of the change from baseline to end of the intervention between the 2 groups, and Cohen’s d for effect size is also reported. The significance level for all statistical tests was set at 0.05.

## 3. Results

**Figure 1:**
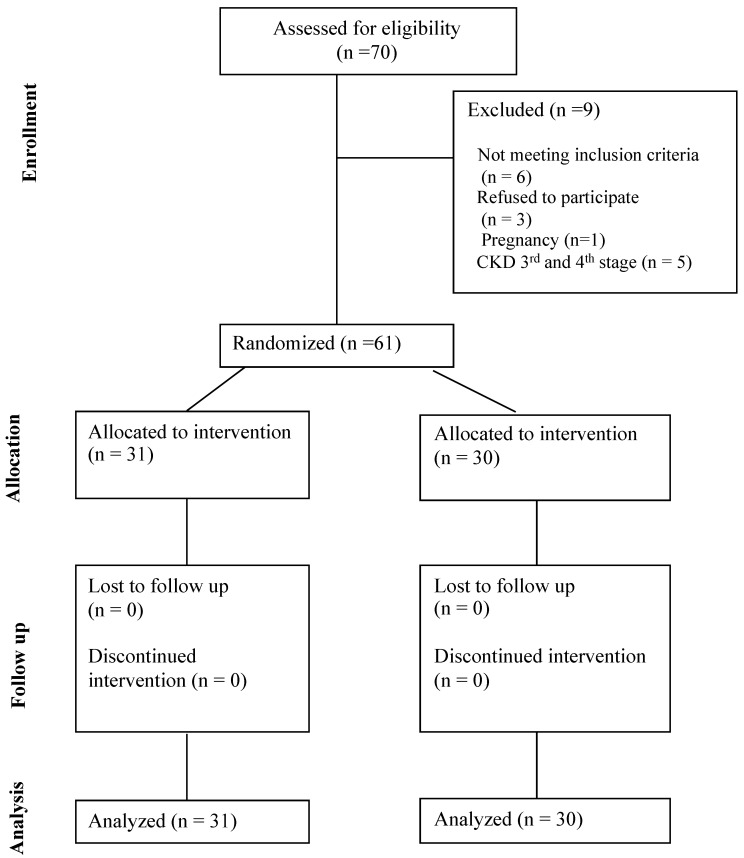
CONSORT Flow Diagram.

From March 2022 (1 March 2022) to September 2022 (30 September 2022), 70 individuals with DMT-1 were checked for eligibility at the Diabetes Outpatient Unit of the 1st Propaedeutic Department of Internal Medicine of University General Hospital of Thessaloniki AHEPA, and 61 individuals were randomized (31 in active group and 30 in control group). Nine individuals in total were not included (six did not fulfill inclusion criteria: one pregnancy, five with CKD third and fourth stage, and three refused to participate). Participant selection is summarized in Figure 1. The last individual was randomized and started the intervention on 30 September 2022 and completed on 30 March 2023 (study completion date). Only 2 people out of 61 reported (one in the active and one in the control group) diarrhea as an adverse event, which was not considered significant to stop the intervention and was resolved after several days. No other adverse event was reported. None of the participants was receiving daily herbal or nutraceutical products.

### 3.1. Participants’ Characteristics

Our sample consisted of 61 individuals with DMT-1, mean age of 48 years old (48.3 ± 14.6), and 26 females (42.6%). Overall participant characteristics are summarized in Table 2. Thirty individuals (49.2%) were on MDI regimen and 31 (50.8%) were on CSII. Thirty-six (59%) were using intermittent glucose monitoring systems, and 25 (41%) were wearing continuous glucose monitoring sensors, which were also connected to the CSII. Six subjects on CSII were using an intermittent glucose monitoring system. Subjects who were on an MDI regimen were all using an intermittent glucose monitoring system. Regarding diabetes complications, 5 (8.2%) individuals had retinopathy, 3 (4.9%) cardiovascular disease and 30 (49.2%) diabetic neuropathy. A total of 36% of our sample were receiving antihypertensive medication, 16% were on antiplatelet drugs, 49% were receiving statins, and 3% on antidepressant agents. Concerning their physical activity, as estimated with IPAQ, 14 (23%) had low, 35 (57.4%) moderate, and 12 (19.7%) high. The groups did not differ significantly on any parameters at baseline (Table 2).

### 3.2. Primary Outcomes

At the end of the intervention, the two groups differed significantly only in serum triglycerides (−10.37 ± 8.13 vs. 7.7 ± 22.7, *p* = 0.049). The intervention group appears to have better glycemic control than the control group after 6 months of taking the supplement. After 6 months of treatment, a significant reduction in the active group was observed in glycated hemoglobin (6.89 ± 0.8 vs. 6.5 ± 0.7, *p* = 0.046) and serum triglycerides (75.4 ± 14.9 vs. 65.03 ± 23.03, *p* = 0.021) (Table 3).

The difference between the groups from baseline to the end of the intervention in the other primary outcomes did not differ significantly between the two groups and within the groups (Table 3). At baseline, the two groups did not differ significantly in any biochemical parameters.

The complete biochemical profile is presented in Supplementary Table S1. There was no difference in the other biochemical variables at baseline and the end of intervention within and between groups (Supplementary Table S1).

### 3.3. Secondary Outcomes

The anthropometric data of the sample at baseline and at the end of the intervention have remained mostly unchanged (Table 4). The same applies to the physical activity and the total daily insulin dose. The difference observed in the control group from baseline to the end of the study in the body composition indices is probably due to a lack of adherence to the preparation instructions for the body composition analysis and may contain an error as no difference in body weight, muscle mass, and waist circumference was observed. It is also possible that the time period of the measurements (usually at the end of the intervention, it was summer) may have influenced the values of the body composition results where the total body water seems lower, probably due to dehydration in the summer months.

Moreover, no difference in dietary intake was observed in the active group, while a significant decrease in fat intake (*p* = 0.019), energy (*p* = 0.037), and mono-unsaturated fatty acids (*p* = 0.002) was observed in the control group (Supplementary Table S2). As mentioned above, no difference in body weight, waist circumference and lipid profile were observed from baseline to the end of the intervention in the control group. Probably, these findings are caused either by reporting bias from the study individuals or because the assessment was conducted during the first 2 weeks and the two last weeks of the study and perhaps was not so representative of the overall fat intake in this group.

Furthermore, there was no difference in glycemic metrics between or within the groups comparing baseline and the end of intervention from glucose monitoring systems data (Table 5). Glucose monitoring data represent only two weeks’ glycemic control at baseline and end of intervention, which may not reflect fully in HbA1c values.

Also, the diabetes distress score (Table 6) improved significantly only in the active group (*p* = 0.044), suggesting an overall improvement in diabetes disease burden in these individuals, but that was not significant enough between the two groups.

Finally, results from Subjective Satiety and Hunger Ratings show a significant reduction between meals in the active group in the hunger dimension compared to the control group (Table 7).

## 4. Discussion

Our main findings show that at the end of the intervention, the two groups differed significantly only in serum triglycerides. In the active group, after 6 months of treatment, glycated hemoglobin was significantly reduced, as well as serum triglycerides.

The other primary outcomes did not differ significantly between and within the groups from baseline to the end of the intervention. No changes were observed in anthropometric measurements, physical activity, diet, and other biomarkers that could affect the results. Diabetes distress score improved significantly only in the active group (*p* = 0.044), suggesting an overall improvement in diabetes disease burden in these individuals, but that was not significantly different enough between the two groups. Thus, from the above, we can consider that the improvement in glycated hemoglobin, serum triglycerides, and diabetes distress score observed in the intervention group is mainly due to the intake of the supplement. Glycemia, reported by CGM data, did not demonstrate any difference within or between the two groups at baseline and at the end of the intervention. However, CGM data represent only two weeks’ glycemic control at baseline and end of intervention and may not fully reflect HbA1c values. This is supported in a recent study by Tozzo et al., where it is shown that longer periods of CGM data correspond more accurately to the average glycemia from HbA1c values since longer periods of CGM data (>26 days) and missing data <10% reduce sensor bias and enhance CGM data accuracy [41].

Our findings are partially in agreement with evidence from recent meta-analyses that saffron has a favorable effect on the glycemic control of individuals with DM. Saffron has shown effectiveness in reducing FBG in individuals with DMT-2 and prediabetes [24,25,42] and in reducing HbA1c only in prediabetes individuals [25], whereas in a meta-analysis by Asbaghi et al. [43] did not show any significant effect in FBG. The same meta-analysis (6 studies) that took into consideration individuals with and without DMT-2 showed that saffron supplementation produced a significant reduction in triglycerides and total cholesterol and increased HDL levels but had a non-significant effect on LDL cholesterol [43]. In individuals with DMT-2, 100 mg/day of saffron for 8 weeks significantly reduced triglycerides, atherogenic index, FBG, and insulin but not HbA1c and other lipid parameters [42]. In this present study, saffron improved HbA1c and decreased serum triglycerides in the active group after 6 months but did not have any improvement in the FBG. It is worth mentioning that the difference in serum triglycerides was also significant between groups and not only within the active group. Of note, the present study is conducted in individuals with DMT-1 whereas all other available studies in the literature are conducted in individuals with DMT-2 or prediabetes [11,24,25,43].

Available evidence suggests that the concentration and combination of the components of saffron inhibit mechanistic pathways more synergistically than its individual bioactive compounds, suggesting the use of the saffron as a combination and not as sole compounds (e.g., crocin or safranal). Rahmani et al. [44] showed that there is a linear dose-dependent relationship between the dose of saffron (mg/d), triglycerides, and cholesterol. Saffron has been proposed for acting hypotensive, hypolipidemic, and antidiabetic through several pathways. Saffron is considered to decrease systolic blood pressure through its vasomodulating effects and anti-inflammatory effects. Hypotensive effects also can be attributed to the blocking of calcium channels and possible interaction with endothelial nitric oxide (NO) [45]. One possible lipid-lowering mechanism is through the reduction of lipid peroxidation factors such as malondialdehyde (MDA) by increasing the action and expression of antioxidant enzymes (such as glutathione reductase activity, superoxide of dismutase and others), preventing phosphorylation of certain protein kinases (e.g., I kappa B kinase-a (IKK) and AMP-activated protein kinase (AMPK)) and reducing the production of reactive oxygen species (ROS) [46]. Another possible mechanism is regulating the expression of growth factors such as tumor necrosis factor-α, adiponectin, and leptin in adipose tissue or fat mass [47]. Potential mechanisms of the effect on the lipid profile may be a potential inhibitory action of saffron and its bioactive ingredients on pancreatic lipase, antioxidant action, increase in the levels of adiponectin, activation of peroxisome proliferator-activated receptor alpha (PPAR-α) and modulation of heat shock proteins [48]. One of the possible mechanisms of the beneficial action of saffron in metabolic control is the antioxidant aspect. Evidence in experimental studies shows that crocin can reduce oxidative stress by decreasing MDA and increasing glutathione [49]. DM is a metabolic disorder that presents with increased oxidative stress due to chronic hyperglycemia [50]. Therefore, we consider that saffron exerts an antioxidant beneficial action in the DM pathway. Also, its antidiabetic action is through the reduction of blood glucose by amplifying glucose uptake into cells, improving insulin signaling in insulin-sensitive tissues (adipose tissue and muscle) and increasing glucose transporter type 4 (GLUT-4) into the cell membranes. These hypoglycemic effects are shown by enhancing GLUT4 translocation into the plasma membrane via the AMPK/ACC pathway [17,51]. Saffron increases antioxidant enzymes (function) reduces ROS production by interfering with ROS-related pathways, thus reduces oxidative stress [46].

Our study has several strengths. To our knowledge, this is the first double-blind, randomized clinical trial investigating the impact of *Crocus sativus* L. extract in people with DMT-1. It is of high importance that the supplement used was a standardized formulation and that the titration of saffron extract was known. It is also worth mentioning that the supplement used was well accepted and tolerated by our study participants. Of note, our glycemic findings are more robust due to continuous glucose monitoring both at baseline and end of intervention. Study duration was one of the major strengths, as all other similar studies have shorter intervention times. The study design was rigorous to ensure compliance and avoid confounding parameters during the intervention through close monitoring of patients and diligent dietary intake capture. Physical activity level assessment at both time points also provided credibility to our findings to ensure that any arising changes are not attributed to physical activity changes.

Our study also has limitations. Glucose monitoring data reflected only two weeks’ glycemic control at baseline and end of intervention. Presumably, collecting data for a longer period of time would be more efficient in understanding and confirming the improvement in glycemic control demonstrated with the HbA1c. Of note, the quantity of saffron extract used, may be insufficient to demonstrate efficacy to other biomarkers. However, we aimed to use a commercial supplement with a standardized formulation to ensure the bioactive ingredients’ proper delivery (bioavailability). Lastly, some differences in dietary intake may have resulted from reporting bias, and we should have aimed to have dietary intake documentation throughout the 6 month period. However, we decided to avoid burdening our participants with more reporting tasks for the study to ensure compliance with the protocol.

Future research should focus on evolving delivery of the saffron bioactives through supplements and developing supplements with a higher proportion of these bioactives. Also, more randomized controlled studies with a more extended period of time need to be conducted in individuals with either DMT-1 or DMT-2 to explore the full potential and safety of saffron supplementation in DM. Should discuss the results and how they can be interpreted from the perspective of previous studies and the working hypotheses. The findings and their implications should be discussed in the broadest context possible. Future research directions may also be highlighted.

## 5. Conclusions

The present study demonstrated that the administration of a probiotic supplement containing saffron extract over a period of six months was well-tolerated by the participants. Furthermore, it was shown to significantly enhance glycemic control and reduce triglyceride levels in individuals with well-controlled DMT-1. These findings suggest that the incorporation of saffron extract into the treatment plan of people with DMT-1 could provide additional benefits to standard treatment, enhancing and improving overall metabolic health that is essential to these individuals. Further research needs to be conducted with newer agents to clarify the extent and frequency of the administration of saffron to exert its beneficial action.

## Figures and Tables

**Table 1:** Formulation of product.

Ingredients	Per Dose (Sachet)	%RDA
Magnesium	75 mg	20
*Lactobacillus rhamnosus*	Min 1 billion CFU	
*Bifidobacterium animalis* subscp. *Lactis*		
*Bifidobacterium breve*		
*Bifidobacterium longum*		
*Fructo-oligosaccharides*	100 mg	
*Crocus sativus* extract	28 mg	
Crocins and safranal	3.5%	

**Table 2:** Participant characteristics at baseline.

Sample	All (*n* = 61)	Active Group (*n* = 31)	Control Group (*n* = 30)	*p*
Gender (M/F)	35 M/26 F (57.4%/42.6%)	17/14	18/12	0.684
Age (years)	48.3 ± 14.6	51.2 ± 15.2	45.4 ± 13.5	0.122
Body weight (kg)	77.2 ± 15.3	77.8 ± 15.3	76.7 ± 15.7	0.793
Body Mass Index (kg/m^2^)	25.9 ± 3.9	26.4 ± 4.1	25.3 ± 3.8	0.253
Waist circumference (cm)	93 ± 16.4	93.7 ± 19.4	92.4 ± 12.9	0.759
Body composition analysis				
Fat mass (kg)	20.7 ± 8.6	22.3 ± 9.4	18.9 ± 7.5	0.133
Fat free mass (kg)	57.2 ± 13	55.8 ± 11.7	58.6 ± 14.3	0.416
Muscle mass (kg)	15.5 ± 5.4	14.9 ± 5.1	16.1 ± 5.8	0.413
Body water (L)	41.7 ± 8	41.1 ± 7.3	42.3 ± 8.8	0.576
DMT-1 duration (years)	25.6 ± 11.1	28.3 ± 11.3	22.8 ± 10.3	0.056
DMT-1 complications	10 (16.4%)	8	2	0.081
Lipid-lowering therapy	30 (49.2%)	15 (48.4%)	15 (50%)	0.900
Antihypertensive medication	22 (36.1%)	15 (48.4%)	7(23.3%)	0.042
Antiplatelet medication	10 (16.4%)	6 (19.4%)	4 (13.3%)	0.525
Antidepressant/Anxiety medication	2 (3.3%)	1 (3.2%)	1 (3.3%)	0.981
Allergies	11 (18%)	3 (9.7%)	8 (26.7%)	0.084
Multiple Daily Injections	30 (49.2%)	19	11	0.074
Continuous subcutaneous insulin infusion	31 (50.8%)	12	19	0.074
Usage of continuous glucose monitoring	25 (41%)	9	16	0.071
Use of intermittent glucose monitoring	36 (59%)	22	14	0.071
Total Daily Dose Insulin (IU)	49.2 ± 25.6	50.8 ± 31.2	47.6 ± 18.6	0.638

Abbreviations: DMT-1: Diabetes Mellitus type 1.

**Table 3:** Primary outcomes for both groups at baseline and end of intervention.

	Active Group				Control Group				
	Baseline	End	D	*p*	Baseline	End	*p*	d	*p*
Fasting Blood Glucose (mg/dL)	127.4 ± 63.3	136.8 ± 48.8	−0.114	0.531	134.9 ± 47.3	147.6 ± 61.6	0.295	−0.195	0.865
Glycated Hemoglobin (%)	6.89 ± 0.8	6.5 ± 0.7	0.374	**0.046**	7.097 ± 0.8	7.1 ± 0.8	0.922	−0.018	0.074
Total Cholesterol (mg/dL)	176.2 ± 30.3	163.3 ± 39.2	0.357	0.056	161.6 ± 26.6	160.7 ± 38.6	0.898	0.024	0.222
HDL cholesterol (mg/dL)	58.9 ± 14.1	61.1 ± 15.2	−0.301	0.104	58.1 ± 11.3	58.4 ± 15.99	0.910	−0.021	0.485
LDL cholesterol (mg/dL)	102 ± 30.6	92.5 ± 24.1	0.339	0.069	88.8 ± 26.6	91.2 ± 28.2	0.579	−0.102	0.079
Triglycerides (mg/dL)	75.4 ± 14.9	65.03 ± 23.03	0.439	**0.021**	73.2 ± 21.5	80.9 ± 44.2	0.345	−0.175	**0.049**
Systolic Blood Pressure (mmHg)	123.1 ± 11.7	124.1 ± 9.5	−0.093	0.619	120.4 ± 14.8	120.7 ± 16.3	0.896	−0.024	0.321
Diastolic Blood Pressure (mmHg)	70.9 ± 11.6	69.4 ± 8	0.022	0.555	68.1 ± 8.1	69.6 ± 8.1	0.395	−0.158	0.931
Pulse Rate	67.7 ± 6.1	66.2 ± 8.0	0.724	0.360	66.9 ± 6.5	66.9 ± 10	0.945	−0.137	0.740

Bold indicates significance.

**Table 4:** Anthropometric measurements.

	Active Group			Control Group			*p*
	Baseline	End	*p*	Baseline	End	*p*	
Body weight (kg)	77.8 ± 15.3	77.9 ± 15.5	0.860	76.7 ± 15.7	76.8 ± 14.9	0.878	0.9
Body Mass Index (kg/m^2^)	26.4 ± 4.1	26.5 ± 4.5	0.744	25.3 ± 3.8	25.4 ± 3.9	0.560	0.821
Waist circumference (cm)	93.7 ± 19.4	94.1 ± 15.8	0.761	92.4 ± 12.9	92 ± 12.7	0.635	0.638
Fat mass (kg)	22.3 ± 9.4	22.9 ± 10.8	0.311	18.9 ± 7.5	20.8 ± 8.6	**0.015**	0.242
Fat free mass (kg)	55.8 ± 11.7	54.9 ± 11.1	0.064	58.6 ± 14.3	56.3 ± 13.6	**0.002**	0.110
Muscle mass (kg)	14.9 ± 5.1	14.3 ± 5.2	0.105	16.1 ± 5.8	15.6 ± 5.9	0.158	0.751
Total body water (L)	41.1 ± 7.3	40.8 ± 6.9	0.373	42.3 ± 8.8	40.7 ± 8.3	**0.002**	0.055
Physical Activity	2659.1 ± 3074.3	2496.9 ± 2912.7	0.835	3466.9 ± 4869.5	2175.4 ± 2725.2	0.225	0.385
Insulin Total Daily Dose (IU)	50.8 ± 31.2	50.1 ± 32.9	0.522	47.6 ± 18.6	47.7 ± 17.4	0.985	0.663

Bold indicates significance.

**Table 5:** Data from glucose monitoring systems.

	Active Group			Control Group		
	Baseline	End	*p*	Baseline	End	*p*
***Time in range***						
Time in range (70–180) (%)	73.1 ± 10.2	74.1 ± 8.9	0.505	65.9 ± 15.9	65.5 ± 15.2	0.875
Time in range (70–180) (min)	1052.4 ± 146.2	1069.0 ± 122.3	0.494	949.8 ± 224.3	947.7 ± 225.9	0.952
***Time above range***						
Time above (181–250) (%)	16.6 ± 6.3	16.9 ± 7.4	0.726	20.9 ± 9	20.4 ± 8.2	0.719
Time above (181–250) (min)	238.8 ± 90.4	243.5 ± 106.9	0.743	300.1 ± 130.5	293.7 ± 117.9	0.734
Time above (>250) (%)	4.4 ± 4.5	4.2 ± 3.1	0.592	8.9 ± 9.6	9.9 ± 11.9	0.474
Time above (>250) (min)	62.7 ± 65	58 ± 41.8	0.241	127.2 ± 138.2	143.4 ± 171.6	0.461
***Time below range***						
Time below (54–69) (%)	4.7 ± 4.9	3.9 ± 2.7	0.234	3.6 ± 2.3	3.4 ± 2.9	0.701
Time below (54–69) (min)	67.9 ± 69.5	55.4 ± 37.3	0.462	51.4 ± 32.9	48.4 ± 40.2	0.625
Time below (<54) (%)	1.2 ± 2.1	1 ± 1	0.490	0.8 ± 1.3	0.7 ± 0.9	0.601
Time below (<54) (min)	17.1 ± 28.2	14.4 ± 14.5	0.277	11.9 ± 19.3	10 ± 12.6	0.605
GMI	6.7 ± 0.4	6.7 ± 0.4	0.602	7 ± 0.7	7.2 ± 1	0.187
Average Sensor Glucose	142 ± 15.9	143 ± 15.1	0.565	155.6 ± 27.3	157.8 ± 30.7	0.582
Average Blood Glucose	139.4 ± 17.9	147.7 ± 25.5	0.329	158.6 ± 34.2	157.9 ± 30.9	0.921
Coefficient of variation (CV)	36.1 ± 7.1	35.5 ± 7.6	0.325	36.5 ± 5.7	35.7 ± 7.6	0.548
Total Carbohydrates consumed (g)	134.4 ± 59.4	123.4 ± 61.9	0.535	174.4 ± 92.6	187.1 ± 107.5	0.140
Total daily dose (IU)	50.8 ± 31.2	50.1 ± 32.9	0.522	47.7 ± 18.6	47.7 ± 17.4	0.985
Insulin bolus units (IU)	27.7 ± 25	26.4 ± 26.3	0.157	24.8 ± 10.9	24.8 ± 9.2	0.997
Insulin basal units (IU)	22.6 ± 11.5	23.8 ± 11.3	0.053	22.9 ± 10.2	22.9 ± 10.8	0.970
Bolus ratio (%)	53.3 ± 10.7	50.5 ± 11.2	0.006	52.3 ± 9.6	52.8 ± 10.5	0.738
Basal ratio (%)	46 ± 11.3	49.5 ± 11.2	0.020	47.7 ± 9.6	47.2 ± 10.5	0.738
Use of active sensor	87.9 ± 14.1	83.7 ± 17.8	0.281	88.2 ± 11	90.2 ± 7.4	0.327
GRI	35.3 ± 18.7	32.5 ± 10.8	0.314	42 ± 19.7	42.5 ± 19	0.864
Events of extended hypoglycemia	2.6 ± 4.8	1.6 ± 1.9	0.222	1.4 ± 2.1	1.3 ± 1.6	0.822
Events of extended hyperglycemia	1.9 ± 2.2	1.6 ± 1.3	0.315	3.8 ± 3.6	3.7 ± 4.1	0.955

Abbreviations: GMI: Glucose management index, GRI: Glycemic Risk Index.

**Table 6:** Diabetes distress score (DDS).

	Active Group				Control Group				Change between the Groups
	Baseline	End	d	*p*	Baseline	End	d	*p*	
Total DDS score	3.3 ± 0.8	3.2 ± 0.6	0.377	**0.044**	3.2 ± 0.7	3.2 ± 0.7	0.332	0.079	0.862

Bold indicates significance.

**Table 7:** Subjective Satiety and Hunger Ratings of individuals.

	Dimension	Active Group	Control Group	*p*
Did you feel less hungry before meals?	Hu	22.2%	3.8%	**0.048**
Did you feel satiated more quickly during meals?	Sa	40.7%	34.6%	0.646
Did the product reduce your need for snacking between meals?	Sn	14.8%	23.1%	0.442
Did the product help reduce your need for snacking related to mood?	Sn	19.2%	7.7%	0.223
Did the product reduce your need for sweet snacks away from meals?	Sn	25.9%	11.5%	0.181
Did the product reduce your need for fatty snacks away from meals?	Sn	14.8%	15.4%	0.626

Abbreviations: Hu: Hunger, Sa: Satiety, Sn: Snacking. Bold indicates significance.

## Data Availability

Data are available upon request for legal and privacy reasons.

## Supplementary Materials

The following supporting information can be downloaded at https://www.mdpi.com/article/10.3390/nu16132089/s1. Table S1. Laboratory results for both groups at baseline and end of intervention. Table S2. Dietary intake in baseline and end of intervention for both groups.
