# Supplementary material for: Effect of Crocus sativus Extract Supplementation in the Metabolic Control of People with Diabetes Mellitus Type 1: A Double-Blind Randomized Placebo-Controlled Trial
Source: Nutrients. 2024 Jun 29;16(13):2089. doi: 10.3390/nu16132089 (PMC11243156; doi:10.3390/nu16132089)
Supplement: Supplementary file 1 [file nutrients-16-02089-s001.zip › nutrients-3062368-supplementary.pdf]

Supplementary Material

Effect of crocus sativus extract supplementation in the metabolic control of people with Diabetes Mellitus type 1: A double-blind randomized placebo-controlled trial

Parthena Giannoulaki [1,2,*], Evangelia Kotzakioulafi [2] , Alexandros Nakas [2], Zisis Kontoninas [2] , Eleni Karlafti [2], Polykarpos Evripidou [2], Konstantinos Kantartzis [3,4,5] , Christos Savopoulos [2], Michail Chourdakis [6] and Triantafyllos Didangelos [2]

Table S1. Laboratory results for both groups at baseline and end of intervention.

	**Active Group**			**Control Group**			**p**
	**Baseline**	**End**	**p**	**Baseline**	**End**	**p**	
Urea (mg/dl)	33,6±12,5	32,9±15,2	0,654	29,1±8,9	29,1±6,8	1	0,722
Creatinine (mg/dl)	0,8±0,1	0,9±0,2	0,339	0,8±0,1	0,8±0,1	0,162	0,091
Uric acid (mg/dl)	4,7±1,1	4,7±1,3	0,756	4,3±1,2	4,2±1,1	0,344	0,358
Aspartate Aminotransferase (U/l)	23,5±5,7	22,8±7,3	0,586	22,4±7,9	21,4±5,3	0,383	0,808
Alanine Aminotransferase (U/l)	22,5±8,8	25,2±15,8	0,216	21±11,2	20,3±8,7	0,624	0,191
Gamma-glutamyl transferase (U/l)	15,1±10,4	16,5±9,5	0,135	14,9±8,5	18,3±21,4	0,290	0,532
Alkaline Phosphatase (U/l)	78,7±22	81,4±27,1	0,188	81,5±30,6	78,5±23,1	0,203	0,067
Potassium (K) (mmol/L)	4,5±0,4	4,6±0,4	0,155	4,4±0,3	4,4±0,4	0,930	0,381
Sodium (Na) (mmol/L)	140,4±1,6	140,3±1,7	0,855	140±2,1	139,4±2,4	0,070	0,227
Calcium (mg/dl)	9,3±0,6	9,4±0,5	0,238	9,4±0,5	9,3±0,3	0,157	0,066
Thyroid Stimulating Hormone (μIU/ml)	2,7±1,3	2,6±1,6	0,824	3,4±6,5	2,2±1,3	0,102	0,132
C-Reactive Protein (mg/dl)	0,5±1,3	0,3±0,2	0,383	0,3±0,1	0,3±0,3	0,079	0,247
Red Blood Count (M/μL)	4,8±0,6	4,8±0,6	0,396	4,9±0,6	5±0,7	0,136	0,085
MCV (fL)	85,7±6,2	86,3±5,9	0,055	84,3±7,5	84,6±7,2	0,441	0,501
WBC (K/μL)	7,1±2,4	7±1,9	0,681	7±1,7	6,6±1,5	0,071	0,374
Hematocrit (%)	40,8±4,6	41±4,8	0,410	41±4,3	41,6±4	0,444	0,249
Hb (g/dL)	14±1,9	14±1,8	0,783	13,8±1,6	14,2±1,5	0,015	**0,027**
Platelets (K/μL)	278±62,1	276±76,6	0,801	257,1±49,6	259,5±62,2	0,696	0,648
TKE (mm)	12,4±11	11±9	0,067	12,7±13,8	8,5±7,2	0,078	0,246
INR	1±0,1	1,8±4,7	0,335	1±0,1	1±0,1	0,472	0,309
PT (sec)	11,2±0,7	10,6±2	0,151	11,3±0,9	11,2±0,8	0,534	0,240
Total serum proteins (g/dL)	7±0,5	7±0,4	0,949	6,8±0,3	7±0,5	0,030	0,069
Serum Albumin (g/dL)	4,5±0,3	4,5±0,3	0,597	4,5±0,3	4,5±0,2	0,229	0,203
Urine Microalbumin (mg/dl)	7,9±17,7	5,5±6,6	0,409	6,1±5,1	8,2±13,6	0,308	0,193

Table S2 . Dietary intake in baseline and end of intervention for both groups

	Active Group			Control Group			P of change
	**Baseline**	**End**	**p**	**Baseline**	**End**	**p**	
Energy (kcal)	2046.9±592.3	2008.4±555.9	0.424	2414.3±606.4	2261.1±447.9	**0.037**	0.173
Carbohydrates (g)	172.3±74.9	172.5±73.8	0.969	201.7±51.5	203.6±52.04	0.820	0.890
Protein (g)	81.6±25.1	79.2±22.6	0.359	87.9±22.4	89.2±25.7	0.716	0.383
Fat (g)	115.2±34.1	112.6±32.2	0.528	135.1±40.7	120.9±28.7	**0.019**	0.098
Fiber (g)	19.3±4.5	18.8±6.6	0.684	20.8±6.2	21.6±6.8	0.469	0.420
Oligosaccharides (g)	93.9±35.7	92.9±42.9	0.902	106.1±33.1	112.8±40.9	0.298	0.439
Sugars (g)	78.3±65.6	79.6±47.2	0.865	95.7±30.3	90.8±30.7	0.475	0.541
Saturated Fat (g)	31.2±10.8	30.7±9.8	0.662	36.2±11.6	34.9±8.8	0.448	0.673
Mono unsaturated (g)	58.8±18.03	58.1±16.7	0.771	70.1±23.9	58.1±16.9	**0.002**	**0.009**
Poly unsaturated (g)	12.1±3.9	12.7±5.9	0.313	15.4±5.4	14.2±4.1	0.266	0.133
Cholesterol (mg)	265.9±134.9	255.4±112.03	0.532	271.4±83.6	286.6±108.9	0.243	0.231

CONSORT 2010 checklist of information to include when reporting a randomised trial*

Section/Topic	Item No	Checklist item	Reported on page No
**Title and abstract**			
	1a	Identification as a randomised trial in the title	1
	1b	Structured summary of trial design, methods, results, and conclusions (for specific guidance see CONSORT for abstracts)	1
**Introduction**			
Background and objectives	2a	Scientific background and explanation of rationale	2-3
	2b	Specific objectives or hypotheses	3
**Methods**			
Trial design	3a	Description of trial design (such as parallel, factorial) including allocation ratio	3
	3b	Important changes to methods after trial commencement (such as eligibility criteria), with reasons	N/A
Participants	4a	Eligibility criteria for participants	3
	4b	Settings and locations where the data were collected	3-4
Interventions	5	The interventions for each group with sufficient details to allow replication, including how and when they were actually administered	4
Outcomes	6a	Completely defined pre-specified primary and secondary outcome measures, including how and when they were assessed	4-5
	6b	Any changes to trial outcomes after the trial commenced, with reasons	N/A
Sample size	7a	How sample size was determined	3
	7b	When applicable, explanation of any interim analyses and stopping guidelines	N/A
Randomisation:			
Sequence generation	8a	Method used to generate the random allocation sequence	4
	8b	Type of randomisation; details of any restriction (such as blocking and block size)	4
Allocation concealment mechanism	9	Mechanism used to implement the random allocation sequence (such as sequentially numbered containers), describing any steps taken to conceal the sequence until interventions were assigned	4
Implementation	10	Who generated the random allocation sequence, who enrolled participants, and who assigned participants to interventions	4

Blinding	11a	If done, who was blinded after assignment to interventions (for example, participants, care providers, those assessing outcomes) and how	4
	11b	If relevant, description of the similarity of interventions	4
Statistical methods	12a	Statistical methods used to compare groups for primary and secondary outcomes	5
	12b	Methods for additional analyses, such as subgroup analyses and adjusted analyses	N/A
**Results**			
Participant flow (a diagram is strongly recommended)	13a	For each group, the numbers of participants who were randomly assigned, received intended treatment, and were analysed for the primary outcome	5
	13b	For each group, losses and exclusions after randomisation, together with reasons	5-6
Recruitment	14a	Dates defining the periods of recruitment and follow-up	5
	14b	Why the trial ended or was stopped	N/A
Baseline data	15	A table showing baseline demographic and clinical characteristics for each group	6-7
Numbers analysed	16	For each group, number of participants (denominator) included in each analysis and whether the analysis was by original assigned groups	6
Outcomes and estimation	17a	For each primary and secondary outcome, results for each group, and the estimated effect size and its precision (such as 95% confidence interval)	7-10
	17b	For binary outcomes, presentation of both absolute and relative effect sizes is recommended	7-10
Ancillary analyses	18	Results of any other analyses performed, including subgroup analyses and adjusted analyses, distinguishing pre-specified from exploratory	N/A
Harms	19	All important harms or unintended effects in each group (for specific guidance see CONSORT for harms)	5,11
**Discussion**			
Limitations	20	Trial limitations, addressing sources of potential bias, imprecision, and, if relevant, multiplicity of analyses	11
Generalisability	21	Generalisability (external validity, applicability) of the trial findings	10-11
Interpretation	22	Interpretation consistent with results, balancing benefits and harms, and considering other relevant evidence	10-12
**Other information**			
Registration	23	Registration number and name of trial registry	3,12
Protocol	24	Where the full trial protocol can be accessed, if available	N/A
Funding	25	Sources of funding and other support (such as supply of drugs), role of funders	4,12

Citation: Schulz KF, Altman DG, Moher D, for the CONSORT Group. CONSORT 2010 Statement: updated guidelines for reporting parallel group randomised trials. BMC Medicine. 2010;8:18.

© 2010 Schulz et al. This is an Open Access article distributed under the terms of the Creative Commons Attribution License (http://creativecommons.org/licenses/by/2.0), which permits unrestricted use, distribution, and reproduction in any medium, provided the original work is properly cited.

*We strongly recommend reading this statement in conjunction with the CONSORT 2010 Explanation and Elaboration for important clarifications on all the items. If relevant, we also recommend reading CONSORT extensions for cluster randomised trials, non-inferiority and equivalence trials, non-pharmacological treatments, herbal interventions, and pragmatic trials. Additional extensions are forthcoming: for those and for up-to-date references relevant to this check
